# Serum Myostatin Is Reduced in Individuals with Metabolic Syndrome

**DOI:** 10.1371/journal.pone.0108230

**Published:** 2014-09-25

**Authors:** Der-Sheng Han, Yu Chu-Su, Chih-Kang Chiang, Fen-Yu Tseng, Ping-Huei Tseng, Chi-Ling Chen, Kwan-Dun Wu, Wei-Shiung Yang

**Affiliations:** 1 Department of Physical Medicine and Rehabilitation, National Taiwan University Hospital BeiHu Branch, Taipei, Taiwan; 2 Department of Physical Medicine and Rehabilitation, National Taiwan University Hospital, Taipei, Taiwan; 3 Department of Laboratory Medicine, National Taiwan University Hospital, Taipei, Taiwan; 4 Department of Internal Medicine, National Taiwan University Hospital, Taipei, Taiwan; 5 Institute of Biomedical Engineering, National Taiwan University, Taipei, Taiwan; 6 Graduate Institute of Clinical Medicine, College of Medicine, National Taiwan University, Taipei, Taiwan; 7 Graduate Institute of Epidemiology and Preventive Medicine, College of Public Health, National Taiwan University, Taipei, Taiwan; 8 Research Center for Developmental Biology and Regenerative Medicine, National Taiwan University, Taipei, Taiwan; Broad Institute of Harvard and MIT, United States of America

## Abstract

**Aims:**

Myostatin is a negative regulator of skeletal muscle mass and may also modulate energy metabolism secondarily. We aim to investigate the relationship between serum myostatin and the metabolic variables in diabetic (DM) and non-diabetic subjects.

**Materials and Methods:**

A cross-sectional study recruiting 246 consecutive DM patients and 82 age- and gender-matched non-diabetic individuals at a medical center was conducted. The variables of anthropometry and blood chemistry were obtained. Serum myostatin level was measured with enzyme immunoassay.

**Results:**

DM group had lower serum myostatin compared with non-diabetics (7.82 versus 9.28 ng/ml, p<0.01). Sixty-two percent of the recruited individuals had metabolic syndrome (MetS). The patients with MetS had significantly lower serum myostatin than those without (7.39 versus 9.49 ng/ml, *p*<0.001). The serum myostatin level decreased with increasing numbers of the MetS components (*p* for trend<0.001). The patients with higher body mass index, larger abdominal girth, lower high-density lipoprotein cholesterol (HDL-C), and higher triglycerides had lower serum myostatin than those without. The serum myostatin level was independently negatively related to larger abdominal girth, higher triglycerides, and lower HDL-C after adjustment. The odds ratios for MetS, central obesity, low HDL-C, high triglycerides, and DM were 0.85, 0.88, 0.89, 0.85, and 0.92, respectively, when serum myostatin increased per 1 ng/mL, in the binary logistic regression models.

**Conclusions:**

Lower serum myostatin independently associated with MetS, central obesity, low HDL-C, and high triglycerides after adjustment. Higher serum myostatin is associated with favorable metabolic profiles.

## Introduction

Myostatin, a member of the transforming growth factor-β (TGF-β) superfamily, is a novel muscle-secreted biofactor that was demonstrated to modulate growth and differentiation of skeletal muscles [1]. Myostatin is mainly expressed in the skeletal muscles, released into extracellular space and blood circulation to exert its paracrine and endocrine effects [2], [3]. Myostatin-deficient mice had a phenotype not only of increased myogenesis, but also decreased fat mass [4], [5]. On the other hand, administration of recombinant myostatin protein to 3T3-L1 preadipocytes or human mesenchymal stem cells was shown to block their adipogenesis [6], [7], [8]. Furthermore, nude mice expressing high-level of recombinant myostatin had a cachexia-like phenotype with reduction in both muscle and fat [9]. Taken together, these results suggest that myostatin is a negative regulator of skeletal muscle mass in development and regeneration to prevent muscle hyperplasia and hypertrophy. On the other hand, myostatin may reduce fat mass by its direct anti-adipogenic effect or indirectly via increasing muscle mass by lessening its inhibition.

In humans, the roles of myostatin in health and disease are far from clear at present. Previously we proposed an “accelerator-brake model” to explain most reported observations in animals and humans. In this model, the expression level of myostatin reflects the endogenous proliferative activity of skeletal muscle and serves as a brake to restrain excessive muscle growth [10]. Consistent with our model, myostatin was previously reported to decrease with age which may parallel the development of sarcopenic obesity in elderly. In our previous studies, we also found that patients with weakened muscles had lower myostatin levels [10], [11]. In a group of pediatric patients with Pompe disease, the myostatin levels increased with enzyme replacement therapy that improved their muscle pathology [11]. How serum myostatin level is related to various metabolic factors has never been extensively addressed. We hypothesized that patients with diabetes and metabolic syndrome may have lower myostatin levels based on the “accelerator-brake” model. Therefore, we recruited 246 type 2 diabetic patients and 82 age and sex-matched non-diabetic controls to investigate the association of serum myostatin with their anthropometric and metabolic status.

## Materials and Methods

### Human subjects

Two hundred and forty six diabetic patients receiving regular out-patient visits at the Metabolism or Nephrology Special Clinics and eighty two age- and gender-matched non-diabetic controls from the Health Check-up Center in the National Taiwan University Hospital (NTUH) between March 2009 and August 2010 were recruited with written informed consents. The study was approved by the institutional review board of NTUH. Blood pressure in sitting position and anthropometric measurements, including height, weight, and abdominal and hip girth, were obtained. Abdominal girth was measured midway between the lowest rib and the iliac crest with a soft tape on standing posture. Body mass index (BMI) was calculated as weight in kilograms divided by the square of the height in meters.

The diagnosis of type 2 diabetes mellitus (DM) was defined following American Diabetes Association criteria as fasting plasma glucose ≧126 mg/dL or 2-hour postload plasma glucose ≧200 mg/dL in an oral glucose tolerance test [12]. The active medications, classified as insulin, metformin, sulfonylureas, thiazolidinediones (TZD), α-glucosidase inhibitors, dipeptidyl peptidase-4 (DPP-4) inhibitors, anti-hypertensives, HMG-CoA reductase inhibitors, and fibrates were recorded from chart review.

The modified National Cholesterol Education Program Adult Treatment Panel III (NCEP ATP III) criterion with Asian cutoff of waist girth was adopted in this study [13]. The MetS was diagnosed when three or more of the following criteria were present. (1) central obesity: waist girth larger than 90 cm in male, and 80 cm in female; (2) hypertension: systolic blood pressure higher than 130 mmHg or diastolic blood pressure higher than 85 mmHg, or previously diagnosed hypertension; (3) low HDL cholesterolemia: HDL cholesterol lower than 40 mg/dl in male, and 50 mg/dl in female; (4) Impaired fasting glucose (IFG): fasting glucose higher than 100 mg/dl, or previously diagnosed type 2 diabetes; (5) hypertriglyceridemia: triglyceride higher than 150 mg/dl [14].

### Laboratory method

The venous blood sample was drawn after overnight fasting for measuring creatinine, plasma glucose, total cholesterol, low-density lipoprotein (LDL) cholesterol, high-density lipoprotein (HDL) cholesterol, and triglycerides by an auto-analyzer (Hitachi 7250 Special, Hitachi, Tokyo, Japan). Hemoglobin and white blood cell count were assessed by an auto-analyzer (Advia 120, Bayer, Germany). The estimated glomerular filtration rate (eGFR) was calculated with the Modification of Diet in Renal Disease Study 4-parameter equation [15].

eGFR (ml/min/1.73 m^2^) = 186.3×Scr (mg/dl)^−1.154^×age (year)^−0.203^×(0.742 if female), where Scr represents serum creatinine.

### Measurement of serum myostatin

Serum myostatin levels were measured with a competitive immunoassay kits according to the manufacturer’s (Immunodiagnostik AG, Bensheim, Germany) protocol. It measured full-length myostatin peptide with high specificity. The test sensitivity was 270 pg/ml, and the intra- and inter-assay variabilities were less than 10% and 15%, respectively [16]. Briefly, the serum samples was thawed and diluted for 5 folds with provided dilution buffer. Being mixed with competitive myostatin antibody solution, the samples were incubated in coated wells for 2 hours at room temperature. After washing, secondary antibody conjugated with peroxidase was added and incubated for another 1 hour. Then the substrate, 3,3′,5,5′-tetramethylbenzidine, for peroxidase was added. The absorption of each well was read using VersaMAX tunable microplate reader (Molecular Devices, Sunnyvale, CA, USA) at 450 nm against 620 nm as a reference. The four-parameter logistic regression model was employed to calculate the concentrations with OD values [17].

### Statistical analysis

The data were presented as the mean ± standard deviation (SD), unless indicated otherwise. The test of means of demographic and clinical characteristics between MetS and non-MetS groups were performed with the independent t-test. Differences in gender and medication frequencies between the MetS and non-MetS patients were analyzed with the χ^2^ test [18]. The null hypothesis was that the values were not different between MetS and non-MetS groups, and the alternative hypothesis was that the values were different between MetS and non-MetS groups. The trend of the demographic and clinical characteristics in terms of increasing number of MetS components was analyzed with one way ANOVA linear trend test [19]. Linear regression models were performed using myostatin level as the dependent variable; and the independent variables were indicated in the tables and context. In model 1, age, gender, DM, MetS, and eGFR were the independent variables. In model 2, age, gender, central obesity, hypertriglyceridemia, low HDL, hypertension, and impaired fasting glucose were the independent variables. Binary logistic regression models were employed to estimate the odds ratios (ORs) for MetS and related metabolic disorders, including central obesity, hypertension, low HDL cholesterol, hypertriglyceridemia, and DM [18]. The multiple comparison error was corrected by the Bonferroni procedure, which limited the significance levels α to 0.05/N. N was the number of multiple hypothesis testing [20]. The independent variables were serum myostatin with adjustment of age and gender. All the significance levels α were set to 0.05. The statistical tests were performed with SPSS® 11.5 (SPSS Inc. Chicago, Illinois, USA).

## Results

A total of 328 subjects were recruited, and 61% of them were male. The average age was 59.0, and the median age was 60.1 years old. Among them, 246 having diabetes mellitus were designated as DM group, and the remaining 82 without DM were control group. There was no difference in age (59.1±13.9 years vs. 59.0±12.2 years, p = 0.978), gender ratio (61% vs.61%, p = 0.948), and body height (162.2±8.6 cm vs. 161.7±9.2 cm, p = 0.687) between the control and the DM group. Generally speaking, the DM group was heavier and had worse metabolic profiles, including higher BMI, abdominal girth, fasting glucose, HbA1c; LDL cholesterol, TG and lower eGFR and HDL cholesterol. Besides, the DM group also had significantly lower serum myostatin level than the control group (7.82±3.85 vs. 9.28±4.08 ng/ml, Table 1).

**Table 1:** Demographic and biochemistry characteristics of patients with and without metabolic syndrome (MetS).

Variables	Non-diabetics				Diabetics			
	Total	Non-MetS	MetS	*P* value*	Total	Non-MetS	MetS	*P* value*
Demographics								
N	82	66	16		246	58	188	
Age (year)	59.1±13.9	57.1±14.4	67.4±7.5	<0.001	59.0±12.2	57.3±12.4	59.5±12.1	0.222.
Gender (% male)	61	65	44	0.118	61	72	57	0.028
Body height (cm)	162.2±8.6	163.4±8.4	157.1±7.9	0.008	161.7±9.2	162.8±7.7	161.4±9.6	0.266.
Body weight (kg)	63.7±9.5**	63.3±9.5	65.6±9.9	0.393	70.3±13.9	64.6±11.2	72.0±14.2	<0.001
BMI (kg/m^2^)	24.17±2.79**	23.61±2.53	26.47±2.72	<0.001	26.7±4.4	24.2±3.2	27.5±4.4	<0.001
Abdominal girth (cm)	82.3±8.2**	81.5±8.5	85.7±5.6	0.065	93.2±10.7	87.1±9.1	95.1±10.4	<0.001
Hypertension (%)	22**	12	63	0.001	74	39	85	<0.001
IFG (%)	41**	34	68	0.012	79	87	77	0.065
Medication								
Insulin (%)	0	0	0	-	13	19	11	0.145
Metformin (%)	0	0	0	-	73	66	76	0.158
Sulfonylureas (%)	0	0	0	-	67	59	70	0.135
Thiazolidinediones (%)	0	0	0	-	34	22	37	0.026
α-glucosidase inhibitors (%)	0	0	0	-	11	9	12	0.451
Dipeptidyl peptidase-4 inhibitors (%)	0	0	0	-	12	10	13	0.624
Oral hypoglycemic agents (%)	0	0	0	-	90	81	93	0.032
Blood Chemistry								
Creatinine (mg/dL)	0.88±0.24**	0.86±0.19	0.96±0.39	0.317	1.37±0.99	1.45±1.26	1.35±0.88	0.490
eGFR (ml/min/1.73 m^2^)	90.3±21.5**	93.4±20.9	77.5±20.0	0.007	66.9±26.4	68.9±25.5	66.3±26.7	0.517
Fasting glucose (mg/dL)	97.2±13.4**	95.5±12.2	104.2±15.9	0.020	128±38	131±33	128±40	0.598
HbA1c (%)	5.78±0.40**	5.71±0.36	6.15±0.45	0.013	7.16±1.25	7.08±1.55	7.18±1.13	0.619
Total cholesterol (mg/dL)	191±34**	195±36	178±19	0.018	176±39	174±35	177±40	0.670
LDL cholesterol (mg/dL)	118±29**	122±30	104±19	0.007	94.2±30.8	96.6±30.8	93.5±30.9	0.543
HDL cholesterol (mg/dL)	54.0±15.6**	56.5±15.7	43.3±10.0	0.002	44.7±11.3	52.9±10.6	42.2±10.3	<0.001
Triglyceride (mg/dL)	109±62**	92±38	183±85	0.001	163±168	88±32	186±186	<0.001
Myostatin (ng/ml)	9.28±4.08**	9.63±4.09	7.82±3.80	0.111	7.82±3.85	9.32±4.65	7.36±3.44	0.004

*:statistical tests between MetS and Non-MetS.

**:statistical tests between total non-diabetics and total diabetics, p<0.01.

The data were presented as the mean ± SD. BMI: body mass index. IFG: impaired fasting glucose. eGFR: estimated glomerular filtration rate. LDL: low density lipoprotein. HDL: high density lipoprotein.

As to the medication history, 90% of the diabetic patients received oral hypoglycemic agents (OHA) for glycemic control, and 13% of them used insulin. Among those patients taking OHA, 73%, 67%, 34%, 11%, and 12% respectively used metformin, sulfonylureas, TZD, α-glucosidase inhibitors, and DPP-4 inhibitors. According to the modified NCEP ATP III criteria, 76% (188/246) of the diabetic patients were classified as having metabolic syndrome (MetS); however, only 20% (16/82) of non-diabetic controls had MetS. There were more females and more patients taking TZD in the MetS group than those without MetS in DM group (Table 1). In addition to those diagnostic components of the MetS, the diabetic patients with MetS also had significantly higher body weight and BMI than those without MetS. Most interestingly, the DM patients with MetS had lower serum myostatin level than their non-MetS counterparts (7.36±3.44 vs 9.32±4.65 ng/ml). The trend that patients with MetS had lower serum myostatin was also observed in the control group (7.82±3.80 vs 9.63±4.09 ng/dl) (Table 1).

All the individuals were further categorized from 0 to 5 according to the number of the MetS diagnostic components they fulfilled. The trend analyses for the anthropometric and biochemical variables were performed on the ordinal variable– components of MetS. Most variables listed in Table 2 showed significantly positive trends with increasing numbers of the MetS components. Notably, the HDL cholesterol, serum myostatin, and eGFR showed significantly negative trends with increasing numbers of the MetS components (Table 2).

**Table 2:** Demographic and biochemistry characteristics of samples according to the components of metabolic syndrome.

Variables	Components of metabolic syndrome						P for trend*
	0	1	2	3	4	5	
N	22	44	58	91	78	35	
Age (year)	58.1±16.9	52.8±13.9	60.1±10.8	60.2±11.2	61.0±12.7	58.2±12.5	0.177
Gender (male ratio)	0.68	0.75	0.64	0.64	0.49	0.51	0.024
Body weight (kg)	59.8±9.5	63.3±9.4	65.9±10.9	68.3±12.7	72.6±14.3	77.1±14.8	<0.001
BMI (kg/m^2^)	22.1±2.0	23.1±2.7	25.1±2.8	26.0±3.4	28.1±4.8	29.5±4.2	<0.001
Abdominal girth (cm)	76.8±7.2	82.9±8.2	87.7±8.8	90.7±9.0	96.1±10.8	100.1±9.7	<0.001
Hypertension (%)	0	9	45	75	86	100	<0.001
IFG (%)	0	59	80	76	75	80	<0.001
Oral hypoglycemic agents (%)	0	23	64	78	92	91	<0.001
Fasting glucose (mg/dL)	88±10	109±28	122±32	129±39	120±34	130±47	<0.001
HbA1c (%)	5.56±0.15	6.24±0.79	6.96±1.69	7.15±1.07	7.05±1.12	7.35±1.30	<0.001
HDL cholesterol (mg/dL)	60.7±18.5	57.7±12.1	50.4±11.3	45.8±11.9	41.3±8.4	36.7±6.2	<0.001
Triglyceride (mg/dL)	79±26	83±31	99±39	151±110	165±95	319±341	<0.001
Myostatin (ng/ml)	9.31±4.37	9.71±4.15	9.38±4.55	7.56±4.12	7.61±3.04	6.47±2.12	<0.001
Creatinine (mg/dL)	0.84±0.20	1.04±0.69	1.32±1.17	1.23±0.80	1.34±0.83	1.48±1.06	0.002
eGFR (ml/min/1.73 m^2^)	96.2±21.0	89.6±26.0	70.7±23.4	71.7±24.6	64.6±27.8	61.3±26.2	<0.001

*:Linear trend test in one way ANOVA.

The data were presented as the mean ± SD. BMI: body mass index. IFG: impaired fasting glucose. HDL: high density lipoprotein. eGFR: estimated glomerular filtration rate.

In order to dissect the relationship between myostatin and metabolic factors, we then sub-grouped these patients according to the diagnostic components of the MetS, and compared the differences in serum myostatin levels. The serum myostatin level was significantly lower in those patients who had MetS (7.39±3.46 vs 9.49±4.35 ng/ml, p<0.001), impaired fasting glucose (7.94±3.80 vs 8.91±4.26 ng/ml, p = 0.020), higher BMI (7.61±3.84 vs 8.94±3.98 ng/ml, p = 0.002), larger abdominal girth (7.46±3.61 vs 9.39±4.23 ng/ml, p<0.001), lower HDL cholesterol (7.56±2.92 vs 9.07±4.45 ng/ml, p = 0.001), and higher TG (6.93±2.95 vs 8.85±4.23 ng/ml, p<0.001). However, the serum myostatin was not decreased significantly in those patients with elevated blood pressure. Thus, patients with unfavorable profiles in the metabolic parameters had decreased serum myostatin levels.

As to the relationship between chronic kidney disease (CKD) and myostatin, we defined patients according to the criteria proposed by the National Kidney Foundation (NKF), that is eGFR = 60 ml/min/1.73 m^2^, and about 33.7% had CKD [21]. When we further categorized the diabetic patients into five stages by the NKF criteria, those with CKD stage V had higher level of serum myostatin than those with CKD stage I (12.15±5.91, n = 12 vs 7.45±3.88 ng/ml, n = 50). The later CKD stage the patient belonged to, that is more severe, the higher serum myostatin he/she had (p for trend <0.001). This negative correlation between renal function and serum myostatin level still existed after adjustment for age, gender, diagnosis of MetS and DM (Table 3, Model 1).

In order to see the net effect of the correlated variables on myostatin, we then chose age, gender, and the variables representing DM, MetS, and renal function as independent variables in multi-variate linear regression models (Table 3). Serum myostatin was positively related to age and male gender. The diagnosis of MetS and eGRF were independently related to the serum myostatin level after adjusting age and gender; however, the diagnosis of DM was not (Table 3, Model 1). Furthermore, we chose the five diagnostic components as independent variables in linear regression model to see which component was related to serum myostatin level. Serum myostatin was negatively related to central obesity, hypertriglyceridemia, and lower HDL cholesterol significantly, and not related to hypertension and impaired fasting glucose (Table 3, Model 2). Thus, central obesity, serum TG and HDL cholesterol level were significantly related to serum myostatin level, after adjustment of age, gender, hypertension and impaired fasting glucose.

**Table 3:** Regression coefficients β (SE) from multiple linear regression models for serum myostatin.

Variables	Model 1 R^2^_adj_ = 0.166**	Model 2 R^2^_adj_ = 0.174**
Age (year)	0.073 (0.018) p<0.001	0.073 (0.017) p<0.001
Gender (male)	1.092 (0.413) p = 0.009	1.012 (0.456) p = 0.027
Diagnosis of MetS	−2.152 (0.486) p<0.001	
Diagnosis of DM	0.664 (0.575) p = 0.249	
eGFR (ml/min/1.73 m^2^)	−0.019 (0.009) p = 0.042	
Central obesity^1^		−1.287 (0.477) p = 0.007
Hypertriglyceridemia^2^		−1.822 (0.459) p<0.001
Low HDL^3^		−0.943 (0.461) p = 0.042
Hypertension^4^		0.293 (0.456) p = 0.522
Impaired fasting glucose^5^		−0.319 (0.463) p = 0.492

SE: standard error. R^2^_adj_: adjusted coefficient of determination. MetS: metabolic syndrome. DM: diabetes mellitus. eGFR: estimated glomerular filtration rate.

1 central obesity: abdominal girth > = 90 cm in male and 80 cm in female.

2 hypertriglyceridemia: triglyceride > = 150 mg/dl.

3 low HDL: high-density lipoprotein > = 40 mg/dl in male and 50 mg/dl in female.

4 hypertension: systolic blood pressure > = 130 mmHg or diastolic blood pressure > = 85 mmHg.

5 Impaired fasting glucose: fasting glucose > = 100 mg/dl.

Since myostatin was independently related to MetS, we estimated the odds ratio (OR) for metabolic disorders, including MetS, central obesity, low HDL cholesterol, hypertriglyceridemia, and DM, associated with different concentrations of myostatin in logistic regression models. Using metabolic disorders as the dependent variable, and age, gender, and serum myostatin as the independent variables, the ORs for MetS, central obesity, low HDL, hypertriglyceridemia, and DM were 0.85 [95% confidence interval (C.I.) = 0.79–0.90, p<0.001], 0.88 (95% C.I. = 0.82–0.93, p<0.001), 0.89 (95% C.I. = 0.83–0.95, p = 0.001), 0.85 (95% C.I. = 0.79–0.92, p<0.001), and 0.91 (95% C.I. = 0.85–0.97, p = 0.003), respectively, with every 1 ng/ml increase in serum myostatin level. Thus, higher serum myostatin was associated with better risk profile for metabolic disorders.

## Discussion

In this study we demonstrated that the serum myostatin level is lower in the patients with DM, and there was a negative trend between serum myostatin level and the number of MetS components. In the linear regression models, serum myostatin was negatively related to the presence of MetS, larger AG, higher TG, and lower HDL cholesterol significantly after adjusting age, gender, higher BP and impaired fasting glucose. Furthermore, those patients with higher serum myostatin had lower odds for MetS, central obesity, low HDL cholesterol, high TG, and DM after adjustment for age and gender. These data suggest that higher serum myostatin is associated with favorable metabolic profiles.

Recently Brandt et al. reported that type 2 DM patients had significantly higher levels of muscle myostatin mRNA content than the healthy controls by 1.4-fold [22]. They also had slightly elevated plasma myostatin level than the healthy controls by only 1.1-fold, which was significantly only after adjusting age and gender. The association of muscle myostatin mRNA (but not plasma myostatin) with impaired insulin sensitivity, increased triglyceride, and obesity was observed only in the healthy controls, but not in type 2 DM patients. They proposed that other factors could probably overrule the negative effect of myostatin on metabolism in patients with diabetes. Hittel et al. employed Western blot to analyze secreted myostatin concentration from primary myotubes from extremely obese women, and found that higher myostatin level in the conditioned media from myotubes of extremely obese individual [23]. However, this is an in vitro study measuring myostatin in conditioned media, not directly from serum or plasma in a normal physiological state. Like the other members of the TGF-β superfamily, serum myostatin concentration is regulated by complex interactions with many proteins in extracellular matrix [24]. Therefore, intracellular mRNA and secretion in isolated muscle tissues may not faithfully reflect its circulating levels. Brandt et al. recruited only 76 type 2 DM patients, which is about 1/3 of our case number. Besides, they recruited fatter (BMI = 30.2) and younger (53.2 years old in the control group) individuals than our study (BMI = 26.0, 59.1 years old). Furthermore, the average age is significantly different between the control (53.2 years old) and DM group (58.2 years old) in Brandt study. In both our and their regression model, the serum myostatin level is related positively with age and negatively with BMI. Thus, smaller sample size and heterogeneity of their recruited subjects were the probable explanation for the seemingly conflicting results.

Myostatin-deficient mice had a phenotype of increased myogenesis and decreased adipose mass in inguinal, retroperitoneal, epididymal, and parametrial fat [4], [5]. Skeletal muscle-specific myostatin transgenic male mice which over-expressing myostatin had 18% reduction in quadriceps and gastrocnemius fiber cross-sectional area and 66% increase in epididymal fat pad mass [25]. On the other hand, nude mice expressing high-level of recombinant myostatin had a cachexia-like phenotype with reduction in both muscle and fat [9]. Feldman et al. found that myostatin transgenic mice expressing myostatin in adipose tissue had immature adipogenesis resulting in lower body weight, serum TG, and fasting plasma glucose [26]. These results suggest that the major task of myostatin is to regulate muscle mass and the metabolic effect is most likely secondary to the changes in muscle mass. However, excessive spillover of high-level myostatin from the skeletal muscle may also have a direct inhibitory effect on the adipose tissues.

Previously we proposed an ‘accelerator-brake’ model to explain the biological functions of myostatin in most physiological and pathological circumstances [10]. In this model myostatin plays a passive role, like a brake, to help restrict the final muscle mass only in response to the stimulatory signals in muscle development and regeneration (see Figure 1 in reference 10). On the other hand, the accelerator (e.g. the developmental and regeneration signals) is the active determinant of the muscle mass and how much myostatin would be induced. This model fits well with our data in this study and the major findings in laboratory animals. Briefly, multiple metabolic factors associated with increased inflammatory cytokines, insulin resistance, physical inactivity, and resistance to or deficiency of anabolic hormones, may hamper the “accelerator” functions to maintain proper muscular mass [27]. Therefore, myostatin expression as the results of worse metabolic profiles is not expected to elevate.

Serum myostatin is related inversely to renal function in our study. We previously reported that myostatin, a middle-sized molecule, is accumulated in the dialysis patient [10]. The plausible explanation is due to the poorer excretion ability during the process of kidney function loss. In this study, no patient had severe CKD that needed hemodialysis for treatment; thus, the elevation of serum myostatin started before advent of severe CKD. We cannot tell whether low eGFR is associated with myostatin independent of metabolic parameters in this study, since renal function deterioration is usually complicated with metabolic dysfunction. A study recruiting CKD patients without metabolic disorder will delineate the true relationship.

There are some limitations. First, this is a cross-sectional study. Causal relationship cannot be obtained. Future longitudinal experiment, collecting variables on muscle sample, body composition, and serum inflammatory cytokines, will provide solid evidence and more cause-effect role of myostatin in the diabetic patients. Besides, this study suffered from selection bias. The recruited individuals could not represent the general population since they were all from the clinics at a tertiary referring center. Population-based sampling is needed to generalize our results. Furthermore, the ELISA kit we employed detected full-length myostatin, active peptide, and pro-peptide. The myostatin immunoreactivity does not necessarily equal to its bioactivity, since the pro-peptide is a inhibitory molecule to myostatin. Future study using mass-spectrometry could delineate the compositions of these molecules in human serum.

In conclusion, we measured the serum myostatin and blood biochemistry variables in type 2 diabetic patients and their age and gender matched controls, and found that patients with DM had lower serum myostatin level. Lower serum myostatin independently associated with MetS, central obesity, low HDL cholesterol, and high TG after adjustment for age and gender. Myostatin may be used as a biomarker for metabolic disorders, and higher serum myostatin is associated with favorable metabolic profiles.
